# Supplementary material for: Finger fractures: Epidemiology and treatment based on 21341 fractures from the Swedish Fracture register
Source: PLoS One. 2023 Jul 14;18(7):e0288506. doi: 10.1371/journal.pone.0288506 (PMC10348528; doi:10.1371/journal.pone.0288506)
Supplement: Attachment — Submitted filename: Response to reviewers.docx [file pone.0288506.s003.docx]

**Comments to the Author**

1. Is the manuscript technically sound, and do the data support the conclusions?

The manuscript must describe a technically sound piece of scientific research with data that supports the conclusions. Experiments must have been conducted rigorously, with appropriate controls, replication, and sample sizes. The conclusions must be drawn appropriately based on the data presented.

Reviewer #1: Yes

Reviewer #2: Partly

2. Has the statistical analysis been performed appropriately and rigorously?

Reviewer #1: Yes

Reviewer #2: Yes

3. Have the authors made all data underlying the findings in their manuscript fully available?

The [PLOS Data policy](https://eur01.safelinks.protection.outlook.com/?url=http%3A%2F%2Fwww.plosone.org%2Fstatic%2Fpolicies.action%23sharing&data=05%7C01%7Chenrik.alfort%40ki.se%7C3781270f76b243327d1508db3522bb87%7Cbff7eef1cf4b4f32be3da1dda043c05d%7C0%7C0%7C638162195680543705%7CUnknown%7CTWFpbGZsb3d8eyJWIjoiMC4wLjAwMDAiLCJQIjoiV2luMzIiLCJBTiI6Ik1haWwiLCJXVCI6Mn0%3D%7C3000%7C%7C%7C&sdata=005V53UTGeC9qAjDgeQR3HN3qHvmXL8NMUwZcZR6Edo%3D&reserved=0) requires authors to make all data underlying the findings described in their manuscript fully available without restriction, with rare exception (please refer to the Data Availability Statement in the manuscript PDF file). The data should be provided as part of the manuscript or its supporting information, or de posited to a public repository. For example, in addition to summary statistics, the data points behind means, medians and variance measures should be available. If there are restrictions on publicly sharing data—e.g. participant privacy or use of data from a third party—those must be specified.

Reviewer #1: Yes

Reviewer #2: Yes

4. Is the manuscript presented in an intelligible fashion and written in standard English?

PLOS ONE does not copyedit accepted manuscripts, so the language in submitted articles must be clear, correct, and unambiguous. Any typographical or grammatical errors should be corrected at revision, so please note any specific errors here.

Reviewer #1: Yes

Reviewer #2: Yes

5. Review Comments to the Author

Please use the space provided to explain your answers to the questions above. You may also include additional comments for the author, including concerns about dual publication, research ethics, or publication ethics. (Please upload your review as an attachment if it exceeds 20,000 characters)

Reviewer #1: PONE-D-23-02792 - Finger fractures: Epidemiology and treatment based on 21341 fractures from the Swedish Fracture Register
The submitted manuscript describes epidemiology and treatment of finger fractures from the Swedish Fracture Register (SFR) in the years 2015-2019. While it may potentially represent an interesting contribution to epidemiological studies on fractures as a specific medical condition, the main issue of this study in its current form is that there is no clear research questions/hypothesis. With regards to, how should this study contribute to the understanding of finger fractures more broadly? What is the importance of epidemiological fracture data? As a research article, it needs more contextualization.

Below, I offer a number of comments and questions about the meaning of some content.

Thank you for taking your time reviewing our manuscript! Your questions are answered below (marked in red) with references to specific lines in the revised manuscript when relevant.

Keywords. Please remove ‘finger fracture’. Keywords and title should not include the same words.

This is now changed

Introduction
Lines 58-60. The aim and objectives should be placed in the framework of a research paper that offer novel information to fill a knowledge gap. As it stands, the work is declared as descriptive and it is unclear how scholars may benefit from epidemiology of SFR. Why is important having epidemiological studies on finger fractures? Which is the problem the authors may want to contribute with their research? How epidemiology from SFR contribute to the understanding of finger fractures more broadly?
Some of these arguments are marginally reported in Lines 42-44.

Earlier studies of finger fractures are either small or not very detailed. They are not based on the same data and seldom of a large and heterogenous population. This might give a biased picture. By using a standardised national registry with broad coverage and detailed information such as PROMs, we can present more accurate and detailed data on this common condition. The importance of epidemiological research and the benefit of using the SFR is now described in lines 58-62 and with a new reference (18).

Materials and Methods
Line 93. Please specify the program used for statistics.
Line 94. Is the term ‘gender’ used as synonym of ‘sex’?
Line 97. The statistical test reads ‘Kruskal-Wallis’. Please check throughout the manuscript.

Information about the statistics program is now included (line 105), the spelling of Kruskal-Wallis is corrected, and “gender” has been changed to “sex” throughout the manuscript.

Results
Line 103. Caption to Figure 1 should include more details. Caption to figure 1 is now changed
Lines 107-108. ‘The age distribution according to gender is presented in figure 2.’ Please add a comment within the text. Comment about age differences is now added (line 113-114).

Line 117. Table 1 ‘joint engagement’ odd word choice. Changed to “presence of intra-articular fracture” in table 1 and 2.
Line 141. Table 4. Please pay attention to issues of spacing (either side of the = sign) This is now corrected in table 4.

Discussion
Line 160. Which are these ‘previous epidemiological studies’? Please add references. References are added and the text is changed (line 169-170).

Line 174. ‘ankle’ and not ‘ancle’. Corrected in line 183.
Lines 187-191. Why are additional parameters (e.g. sex, age of the patients) not taken into account when comparing with other studies? More contextualization of SFR data is necessary. Finally, consideration of potential limits in comparability of results should be included. The two epidemiological studies discussed in the manuscript (Feehan 2006 and Karl 2015) also uses data that are supposed to cover all finger fractures in their respective region. There is nothing that suggests that the population described in the SFR is much different from theirs. Based on this, we consider registry coverage as the main reason for our divergent incidence numbers. This is now discussed in lines 198-205

Line 198. Juto et al. (2017) is not included within the final references. Reference is now added (nr 23).
Figure 1. 20337+1007 = 21334 and not 21341. Please revise. Numbers checked and changed
Figure 2. Age range 0-99 years but Results section reports 0-101 years. Please revise. The curve continues after the last scale mark (99). The next mark would be 102. We have chosen to not change this.

Reviewer #2: The paper is well written and the data clearly presented.
It would be needed a better review of the state of the art in the introduction, whereas similar studies are only briefly mentioned in lines 186-189.
A better comparison between the current study and previous studies could also benefit and improve data readability for future studies.

Thank you for your valuable comments.

More discussions about earlier studies are added in several sections: lines 42-44 (not changed in this version), 58-62, 169-170 and 198-205.
