# Supplementary material for: Finger fractures: Epidemiology and treatment based on 21341 fractures from the Swedish Fracture register
Source: PLoS One. 2023 Jul 14;18(7):e0288506. doi: 10.1371/journal.pone.0288506 (PMC10348528; doi:10.1371/journal.pone.0288506)
Supplement: Attachment — Submitted filename: Response to reviewers.docx [file pone.0288506.s004.docx]

Reviewer #2: The Authors successfully incorporated the suggested revision. However, a few grammatical (e.g., ‘data’ takes the plural form of a verb or pronoun as ‘datum’ is the singular form) errors still persist in the revised manuscript.

Thank you. The text is now revised, and we hope that all grammatical errors are corrected.

Comments: Figure 2. As per my earlier comment, x-axis values and age range as reported in the main text should match, therefore I suggest the Authors to add the number 102 as an extra mark.

The number 101 has now been added to Figure 2.

Academic Editor Notes: Dear authors, thank you for your contribution. There are some points which should be further addressed before formal acceptance:
1. Upgrade your manuscript to the STROBE guidelines. Readapt carefully the subheadings. State the use of the STROBE guidelines and cite them. Attach the STROBE checklist as supplementary material

Thank you for taking your time to revise our manuscript! Subheadings has been changed were applicable. This study is based on an already existing database (a national quality register), which none of the authors have taken part in creating. Thus, some of the items on checklist were not relevant to this type of study. The checklist with references to pages and lines in the manuscript is attached as supplementary material. On lines 112-113 the use of STROBE is stated, and the citation is added.

2. abbreviation should be clarified at once, then use only the mentioned abbreviation (e.g. PROMs) This has been checked.

3. Divide methods and results into subheadings.

This is now changed

4. PROMs not PROM!

Changed as suggested.

5. PROMs not PROM scores!

Changed as suggested.

6. when you give percentages, you need also to clarify the number of events/observations. For example 20% (20 of 100)

We find that percentage is much more clear way to describe the material rather than using the large figures in this study. We could add the numbers as well, but we strongly believe that this would negatively affect the clarity and readability of the text. Hence, no changes have been done.

7. Use ALWAYS third person and passive voice

This has now been changed.

8. ABSTRACT:
8.1. Add the conclusion!

Abstract has now been divided into subheadings and a short conclusion was added.

9. METHODS:
9.1. Declare in DETAIL that you follow the principles expressed in the Helsinki declarations AND later amendments, the signed consent of patients must be declared.

According to the legal requirements for national quality registries in Sweden there is no signed consent form. Information about the SFR is given to all patients seeking care for fractures and they can at any time opt out. Their data are erased if they wish so. This is now mentioned in lines 60-63 and 116-117. Two new references have also been added about the structure of the SFR (Wennergren 2015 and 2018).

9.2. How you evaluated the fracture classification? This is one of the most important information that must be explained IN DETAIL, and potential limitations must also be acknowledged

We have not evaluated the fracture classification since the AO/OTA classification was chosen by those who designed the SFR. It is a system that is comprehensible, widely recognised, and valid. They discuss this further in references Wennergren 2015 and 2018.

9.3. Report a figure with the classification system you used. Cite the classification system.

There are now three references about the classification system: Newman 1990, Marsh et al 2007 and Meinberg et al 2018 that describes the classification system. Since the AO/OTA classification is well known and easily accessible through the AO foundation we think a figure describing the classification is superfluous and prefer to not add such figure. I figure describing the system would in addition be large and difficult to interpret. At the AO foundation and the SFR websites user-friendly and clickable versions are available.

9.4. Report the therapeutic framework in DETAIL and add a figure of it in the methods

The SFR includes patients seeking care at trauma centres all over Sweden, therapeutic measures might differ between centres, patient groups and types of fractures and information about this is not included in the registry and we cannot report more details.

10. RESULTS:
10.1. Describe in DETAIL the identification process, with the exact excluded and included patients. Add these also to the flowchart.

Patients presenting with a fracture are included in the SFR by the treating physician at each trauma centre. Everyone is included unless they do not want to. Information about the registry is given to all. Information about excluded patients are not registered.

10.2. If you can add some Figures describing your results will be appreciated. These results are a lot of numbers and figures that could help to summarise your findings

It is difficult to summarize out findings in figures. We believe that the most comprehensive summary is made in text and has tried to limit the number of figures and being cautious not to repeat too much. All findings are presented in detail in the tables and the main findings are found in the text.

11. DISCUSSIONS:
11.1. Limitations have not been identified. Please create a paragraph of 250-500 words identifying all possible limitations.

Limitations of the study are discussed in a new section of discussions, line 231-247

12. CONCLUSIONS:
12.1. There are no conclusions in support of your findings. Please elaborate a strong evidence-based conclusion

A conclusion has now been added to the discussion.
