# Supplementary material for: Finger fractures: Epidemiology and treatment based on 21341 fractures from the Swedish Fracture register
Source: PLoS One. 2023 Jul 14;18(7):e0288506. doi: 10.1371/journal.pone.0288506 (PMC10348528; doi:10.1371/journal.pone.0288506)
Supplement: Attachment — Submitted filename: Response to reviewers.docx [file pone.0288506.s005.docx]

Dear Authors,

Just want to ask you for some minimal revisions:

Remove the subheading "objectives" in the introduction section
divide the methods section into subheadings: Study protocol (add all your declarations: Helsinki, STROBE, ethics etc), Eligibility criteria, Outcome assessment, Statistical analysis
divide also the Results into subheadings according to your findings
Remove subheadings in the discussion section
Limitations: discuss the lack of children until 2015 and its possible effect

Improve the scientific language level and use the third person. There are still several points which could be improved

Thank you,
Filippo Migliorini
Editor

Thank you for your fast response and valuable comments! The manuscript is now revised. Subheadings have now been changed according to your suggestions and the lack of children in the SFR is discussed. Third person is used in the text. Please let us now if there is anything else that can be improved! Changes since the last version is marked in yellow.

Best regards,
